# FeiyanHeji multifunctionally inhibits influenza virus via PA protein degradation and RIG-I signaling pathway potentiation

**DOI:** 10.1186/s13020-026-01372-6

**Published:** 2026-04-22

**Authors:** Tongtong Cao, Wenqiang Sun, Yan Hu, Chang Liu, Yue Hou, Jing Shu, Ningning Zhang, Qiang He, Wenxian Yang

**Affiliations:** 1 Department of Traditional Chinese Medicine, Beijing Children’s Hospital, Capital Medical University, National Center for Children’s Health, Beijing, 100045 China; 2 Beijing Institute of Ophthalmology, Beijing Tongren Eye Center, Beijing Tongren Hospital, Capital Medical University, Beijing Ophthalmology & Visual Sciences Key Laboratory, Beijing, 100730 China; 3 State Key Laboratory of Biopharmaceutical Preparation and Delivery, Institute of Process Engineering Chinese Academy of Sciences, Chinese Academy of Sciences, Beijing, 100190 China

**Keywords:** Traditional Chinese medicine, Network pharmacology, IAV, PA, Innate immunity

## Abstract

Influenza virus infection can lead to high mortality and severe disease complicated by secondary bacterial pneumonia. FeiyanHeji (FyHj) is a traditional Chinese medicine (TCM) mixture used clinically to treat pneumonia caused by influenza, although its mechanism of action remains unclear. We first conducted a network pharmacology analysis of the ten components in FyHj to predict their potential interactions with influenza-related targets. GO and KEGG analyses revealed significant enrichment in the inflammatory response, ubiquitin protein binding, RNA virus-related pathways, and the RIG-I signaling pathway, suggesting these may be involved in the anti-influenza process. Further experimental validation demonstrated that FyHj inhibits viral replication through two mechanisms: (1) promoting the degradation of polymerase acidic (PA) protein by interfering with UBP11-mediated K48-linked deubiquitination, and (2) enhancing antiviral innate immunity by upregulating the RIG-I/MAVS signaling pathway and stabilizing MAVS through inhibition of TRIM25-mediated K48-linked ubiquitination of MAVS. Finally, FyHj was observed to reduce lung viral load and inflammation in mice infected with influenza A virus (IAV). In conclusion, FyHj decoction elicits potent antiviral effects through multiple mechanisms, providing a mechanistic basis for the clinical application of FyHj and highlighting its potential as a source for multi-target anti-influenza therapies.

**Supplementary Information:**

The online version contains supplementary material available at 10.1186/s13020-026-01372-6.

## Highlights

1. FyHj facilitates the degradation of viral PA protein by promoting its K48-linked ubiquitination via UBP11.
2. FyHj enhances antiviral innate immunity by activating the RIG-I/MAVS pathway and stabilizing MAVS through inhibition of TRIM25-mediated K48-linked ubiquitination.
3. FyHj treatment effectively reduces lung viral load and mitigates inflammation in a murine model of influenza A virus infection.

**Supplementary Information:**

The online version contains supplementary material available at 10.1186/s13020-026-01372-6.

## Introduction

Influenza A virus (IAV) is a major respiratory pathogen responsible for recurring seasonal epidemics and occasional pandemics, posing a serious threat to global health. Severe IAV infection often leads to viral pneumonia and acute respiratory distress syndrome and can be further complicated by secondary bacterial infections, contributing to high morbidity and mortality rates [1–3]. During infection, IAV replicates within alveolar epithelial cells, causing extensive cell damage and triggering the host's innate immune response. Viral RNA is recognized by the cytoplasmic pattern recognition receptor retinoic acid-inducible gene I (RIG-I), which activates mitochondrial antiviral signaling protein (MAVS) to induce the production of type I interferons (IFNs) and antiviral cytokines [4, 5]. Enhancing host antiviral immunity while suppressing viral replication is therefore a promising therapeutic strategy for influenza-induced pneumonia.

Currently, several classes of antiviral agents are used in the clinical management of influenza. Neuraminidase inhibitors such as oseltamivir, zanamivir, and peramivir prevent viral release from infected cells, whereas M2 ion channel inhibitors such as amantadine and rimantadine block viral uncoating; however, the widespread emergence of drug-resistant viral strains has significantly reduced their efficacy [6–8]. More recently, RNA polymerase inhibitors have been developed to target the viral replication machinery. The influenza virus RNA polymerase complex—composed of polymerase acidic (PA), polymerase basic protein 1 (PB1), and polymerase basic protein 2 (PB2)—is essential for viral transcription and replication [9]. Among these, the PA subunit functions as an endonuclease, initiating viral mRNA synthesis by cleaving host pre-mRNA caps. Baloxavir marboxil, the first FDA-approved PA endonuclease inhibitor, effectively blocks viral mRNA transcription and exhibits broad-spectrum activity against influenza A and B viruses [10, 11]. However, its therapeutic window is limited to early infection stages, and viral resistance remains a significant concern [12]. Beyond direct-acting antivirals, host-directed therapies have emerged as a complementary strategy, aiming to enhance antiviral innate immunity or modulate host inflammation. In particular, the RIG-I/MAVS signaling pathway is a critical component of the innate antiviral response, yet few clinically available agents have been developed to activate this axis pharmacologically [13, 14]. Therefore, the discovery of novel therapeutics capable of both inhibiting viral replication and enhancing host immunity represents a significant unmet need in influenza treatment.

Traditional Chinese medicine (TCM) offers a rich resource for discovering multi-target antiviral agents with both virus-suppressing and immune-regulating properties [15, 16]. During the COVID-19 pandemic, TCM formulations such as “Maxingshigan Decoction” demonstrated notable efficacy in alleviating respiratory symptoms and reducing disease severity [17–19]. FyHj, a hospital-prepared TCM formula developed at Beijing Children’s Hospital and derived from “Maxingshigan Decoction,” has been widely used for decades in the clinical treatment of pediatric pneumonia. FyHj contains ten herbal components—Mahuang (Ephedrae Herba), Xingren (Armeniacae Semen Amarum), Shigao (Gypsum Fibrosum), Gancao (Glycyrrhizae Radix et Rhizoma), Jinyinhua (Lonicerae Japonicae Flos), Lianqiao (Forsythiae Fructus), Yujin (Curcumae Radix), Qingdai (Indigo Naturalis), Geke (Meretricis Concha), and Zisuzi (Perillae Fructus)—and has shown promising effects in reducing airway inflammation and modulating immune function. However, the molecular mechanisms underlying its anti-influenza activity remain poorly understood. In this study, we combined network pharmacology and experimental validation to elucidate the antiviral mechanism of FyHj. We demonstrate that FyHj suppresses IAV replication by promoting PA protein degradation through inhibition of UBP11-mediated K48-linked deubiquitination, while simultaneously enhancing RIG-I/MAVS-mediated antiviral signaling by stabilizing MAVS. These findings provide new mechanistic insight into FyHj’s therapeutic efficacy and suggest its potential as a source of novel anti-influenza drug candidates.

## Materials and methods

### Network pharmacology data processing

#### Collection of chemical components of TCM and prediction of targets

Active components of TCM were retrieved from the TCMSP database using the criteria of oral bioavailability (OB) ≥ 30% and drug-likeness (DL) ≥ 0.18. Active components and their targets were identified and screened. The obtained targets were calibrated using the UNIPROT database (https://www.uniprot.org/), non-human genes were removed, and invalid duplicates were deleted to obtain standardized gene names. For TCMs not included in the TCMSP database, components and targets were searched in the HERB database.

#### Collection of influenza-related targets

Using the keywords "influenza," targets related to influenza were obtained from the GeneCards, TTD, and CTD databases. All targets from the three databases were integrated into Excel, and duplicate genes were removed. Gene information was then corrected using the UniProt database.

#### Construction of the drug-target-disease protein interaction network

The selected TCM drug targets and influenza-related targets were summarized, and common targets were identified. A Venn diagram was generated using an online platform (http://bioinfogp.cnb.csic.es/tools/venny/index.html). The common gene targets were uploaded to the STRING database to construct a protein–protein interaction (PPI) network. The species was set to "Homo sapiens," and the minimum interaction score was set to 0.4 to ensure reliability. Other parameters remained default. The TSV file format was saved and imported into Cytoscape for visualization. The CytoHubba plugin was used to evaluate the network topology and identify core targets of TCM for influenza treatment.

#### GO and KEGG pathway enrichment analysis

The common genes between drug and disease were uploaded to the DAVID database. OFFICIAL_GENE_SYMBOL was selected as the identifier, and the species was set to Homo sapiens. Gene Ontology (GO) functional annotation was performed to assess the roles of target proteins in biological processes (BP), cellular components (CC), and molecular functions (MF). KEGG pathway enrichment analysis was conducted to elucidate the signaling pathways involved. The top 10 entries for BP, CC, and MF in GO and the top 30 pathways in KEGG were selected as the primary enriched processes and pathways, predicting the mechanism of TCM in treating influenza.

### Cells and virus

The human embryonic kidney cells (HEK293 T), human lung alveolar epithelial (A549) cells, and Madin-Darby canine kidney (MDCK) cells (all from ATCC) were maintained in Dulbecco’s modified Eagle’s medium (DMEM, Invitrogen) with 10% fetal bovine serum (FBS, Gibco) at 37 °C and 5% CO_2_. The influenza virus A/WSN/1933 (H1N1) strain was rescued using a 12-plasmid reverse genetic system (provided by Dr. Ye, Institute of Microbiology, Chinese Academy of Sciences) and propagated in 9-day-old embryonated chicken eggs (Merial, Beijing).

### Drug preparation

FyHj was provided by Beijing Children’s Hospital. The equivalent dose for rats was 1.35 g/100 g of FyHj, calculated based on the body surface area ratio between humans and rats, and served as the basal dose. This basal dose corresponds to three times the clinical dose for adults. FyHj was orally administered to rats once daily at 9:00 A.M. for 12 days. Control group rats received an equal volume of normal saline. Food was restricted for 12 h before administration, but water was available ad libitum. Rats were anesthetized with ether, and blood samples were collected aseptically from the heart 1.5 h after the final administration. Blood samples were kept at 4 °C for approximately 4 h to allow coagulation, then centrifuged at 3500 rpm for 15 min. Supernatants from the same group were pooled, inactivated at 56 °C for 30 min, sterilized by filtration through 0.22 µm filters, and stored at − 70 °C. In this study, the processed FyHj-SER was added to the cell culture medium at a final concentration of 5% v/v.

### Animals

Specific-pathogen-free (SPF) BALB/c female mice, weighing 16–18 g, were purchased from the Jackson Laboratory. Mice were maintained under SPF conditions in the animal facility of the Institute of Microbiology. They were fed a standard laboratory diet and provided water ad libitum. All animal experiments were approved by the Research Ethics Committee of the Chinese Academy of Sciences and followed the Beijing Laboratory Animal Welfare and Ethical Guidelines.

### Mouse inoculation and anti-viral treatment

The mice were intranasally infected with 2 MLD_50_ of mouse-adapted H1N1 virus in a volume of 50 μL. Groups of mice were orally administered 1.35g/100 g/day of FyHj solution respectively. The control animals were treated with the normal saline only. The drug was administered twice a day (at 12 h intervals) for 5 days.

### qRT-PCR

RNA from tissue or cells were homogenized with TRIzol, and RNA was isolated according to the manufacturer’s instructions. RNA levels were measured using the One Step PrimeScript RT-PCR Kit (RR064B, Takara) on a CFX96 real-time PCR system (Bio-Rad). Specific primer information is shown in Table 1 in the supplementary material.

**Table 1:** List of qRT-PCR primer sequences

Genes	Forward primers	Reverse primers
*IFNB1*	5′-AACTGCAACCTTTCGAAGCC-3′	5′-TGTCGCCTACTACCTGTTGTGC-3′
*IFNA*	5′-TCAGCACAAAGGATTCATCTG-3′	5′-TCAGCACAAAGGATTCATCTG-3′
*IFIT1*	5′-TTCGGAGAAAGGCATTAGA-3′	5′-TCCAGGGCTTCATTCATAT-3′
*IFIT2*	5′-TCATTTTGCATCCCATAGGAGGTT-3′	5′-GACTTTGGTCCCCCAGCTTT-3′
*GAPDH*	5′-TTGTCTCCTGCGACTTCAACAG-3′	5′-GGTCTGGGATGGAAATTGTGAG-3′

### Subcellular fractionation

Cells (5 × 10^7^) infected with IAV or PBS and lysed by dounce homogenization buffer (ApplyGen). The homogenates were then centrifuged at 800*g* for 5 min twice. The supernatants were centrifuged at 12,000*g* for 10 min to precipitate mitochondria. The supernatants from this step (cytoplasm fraction) were also collected. The precipitate fraction was washed with 0.2 mL of homogenization buffer, centrifuged at 12,000*g* for 10 min, and collected as the mitochondrial fraction.

### RNA interfering

Cells were transfected with siRNA for UBP11 knockdown or plasmid for UBP11 overexpression using Lipofectamine Transfection Reagent (Invitrogen) as per manufacturer’s protocol. The transfection media was replaced with fresh culture media at 4–6-h post-transfection. Control and UBP11 siRNA were synthesized in Guangzhou RiboBio Co., Ltd. The siRNA sequence pool was given as follows: Human UBP11 (1) GCGCACAGCUGCAUGUCAU; (2) GAGAAAGCACUGGUAUAAGC; (3) GGACCGUGAUGAUAUAUCUUC; control (1) UGGUUUACAUGUCGACUAA; (2) UGGUUUACAUGUUGUGUGA; (3) UGGUUUACAUGUUUUUCUGA.

### Statistical analysis

In vitro cell culture experiments were performed independently in triplicate, and the data are presented as the mean ± standard deviation (SD). GraphPad Prism (version 9.0.2) software was used to present the statistical analysis data. Data are analyzed using Student’s *t*-test (two-tailed). A *p* value less than 0.05 was regarded as a statistically significant difference.

## Results

### Prediction of targets of TCM in treating influenza

In this study, a threshold was established during target selection to enhance data reliability and validity. Based on screening criteria including OB ≥ 30%, DL ≥ 0.18, and excluding components without identified targets, the TCMSP database yielded the following active compounds: 88 from Gancao, 17 from Jinyinhua, 19 from Lianqiao, 14 from Mahuang, 19 from Qingdai, 12 from Xingren, 3 from Yujin, and 13 from Zisu. Additionally, 1 compound was identified from Shigao and 2 from Geke using the herb database. After integrating and deduplicating the targets of all compounds, a total of 641 potential therapeutic targets of traditional Chinese medicine were identified.

A total of 594 influenza-related targets were retrieved from the CTD database and 41 from the TTD database. Using a relevance score greater than 2 as the selection criterion, 1,435 influenza-associated targets were obtained from the GeneCards database. Integration of targets from all three databases yielded a comprehensive set of 1757 influenza-related targets.

**Fig. 1:**
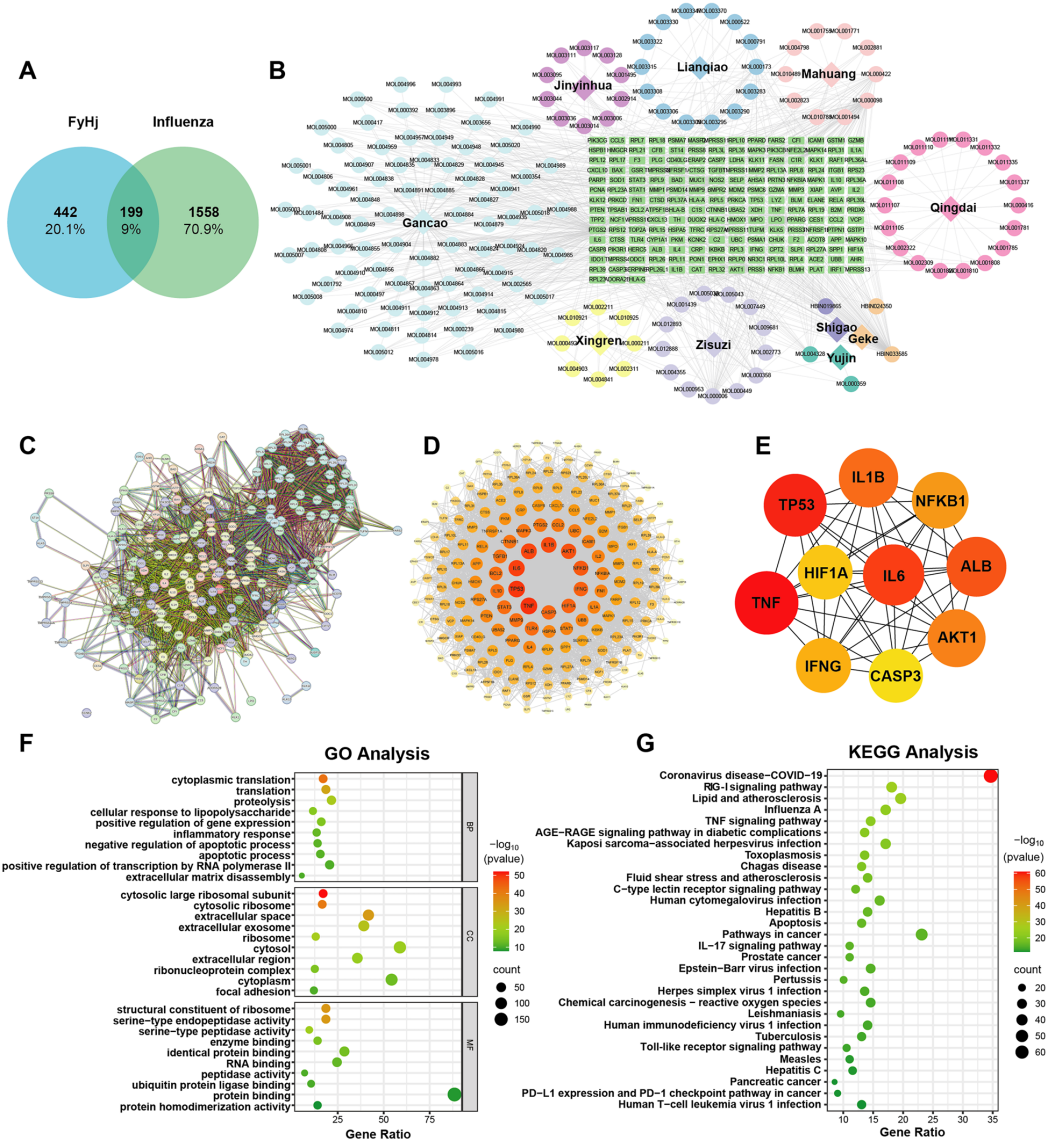
The network pharmacology analysis of FyHj. **A** Venn diagram of FyHj TCM targets and influenza targets. **B** Drug-component-target network map. **C** The protein–protein interaction (PPI) map obtained from the STRING database. **D** The PPI map obtained by Cytoscape software. **E** The PPI map of the main target. Using the CytoHubba plugin, the top 10 targets were screened based on the degree value as the primary targets. **F** The GO analysis of common targets of FyHj and influenza. The common targets involved in the biological processes (BP), Cellular Component (CC) and molecular functions (MF) of influenza were analyzed in the *David* database. A total of 741 GO items were retrieved, including 561 BP, 89 CC, and 91 MF. Select the top 10 items of each category for visualization based on the *p*-value. **G** The KEGG analysis of common targets of FyHj and influenza. A total of 164 signaling pathways enriched by the KEGG pathway were analyzed. The top 30 enriched pathways were represented by bubble plots, with the horizontal axis indicating the amount of gene enrichment, the size of the bubbles representing the number of genes, and the color depth representing the *p*-value

A Venn diagram of the "drug–disease" targets identified 199 potential therapeutic targets of TCM for influenza treatment (Fig. 1A). These targets were visualized using Cytoscape software to construct a "drug–component–target" interaction network (Fig. 1B and Supplementary Data 1). Analysis of the TCM and influenza targets identified potential components involved in anti-influenza mechanisms, warranting further in-depth investigation.

### Construction of the common target PPI network

The common targets obtained from the Venn diagram were imported into the STRING database, resulting in a PPI network comprising 199 protein nodes and 4,013 edges (Fig. 1C). The network was further visualized using Cytoscape 3.8.2 (Fig. 1D), where larger node sizes and darker colors reflect higher centrality and greater importance within the network. Using the Cyto-Hubba plugin, the top 10 targets were ranked based on their degree values and identified as core targets (Fig. 1E). This study concludes that the main targets of TCM in treating influenza include factors related to inflammation such as TNF, TP53, IL-6, ALB, and IL1B.

### GO and KEGG enrichment analysis

To investigate the potential molecular mechanisms underlying the treatment of influenza by TCM, common targets associated with the biological processes and molecular functions of influenza were analyzed using the DAVID database. A total of 741 GO terms were identified, comprising 561 biological processes (BP), 89 cellular components (CC), and 91 molecular functions (MF). The top 10 terms in each category were selected based on their *P* values for visualization purposes (Fig. 1F). Additionally, 164 signaling pathways were enriched through KEGG pathway analysis. The top 30 enriched pathways were visualized using bubble charts, where the horizontal axis represents the degree of gene enrichment, the size of each bubble reflects the number of genes involved, and the color intensity corresponds to the *P* value (Fig. 1G). In summary, the GO and KEGG analyses revealed that the inflammatory response (BP), ubiquitin protein binding (MF), RNA virus-related pathways, and RIG-I signaling pathways were significantly enriched and might be involved in the process of TCM's anti-influenza effect. Because the main ingredients of FyHj formula are derived from the Maxingshigan Decoction, and numerous studies have shown that it can exert anti-inflammatory effects. In this research, we mainly focused on the other three possible anti-viral processes (ubiquitin protein binding, RNA virus-related pathways, and RIG-I signaling pathways).

### FyHj-SER can inhibit the influenza by promoting the degradation of the PA protein

**Fig. 2:**
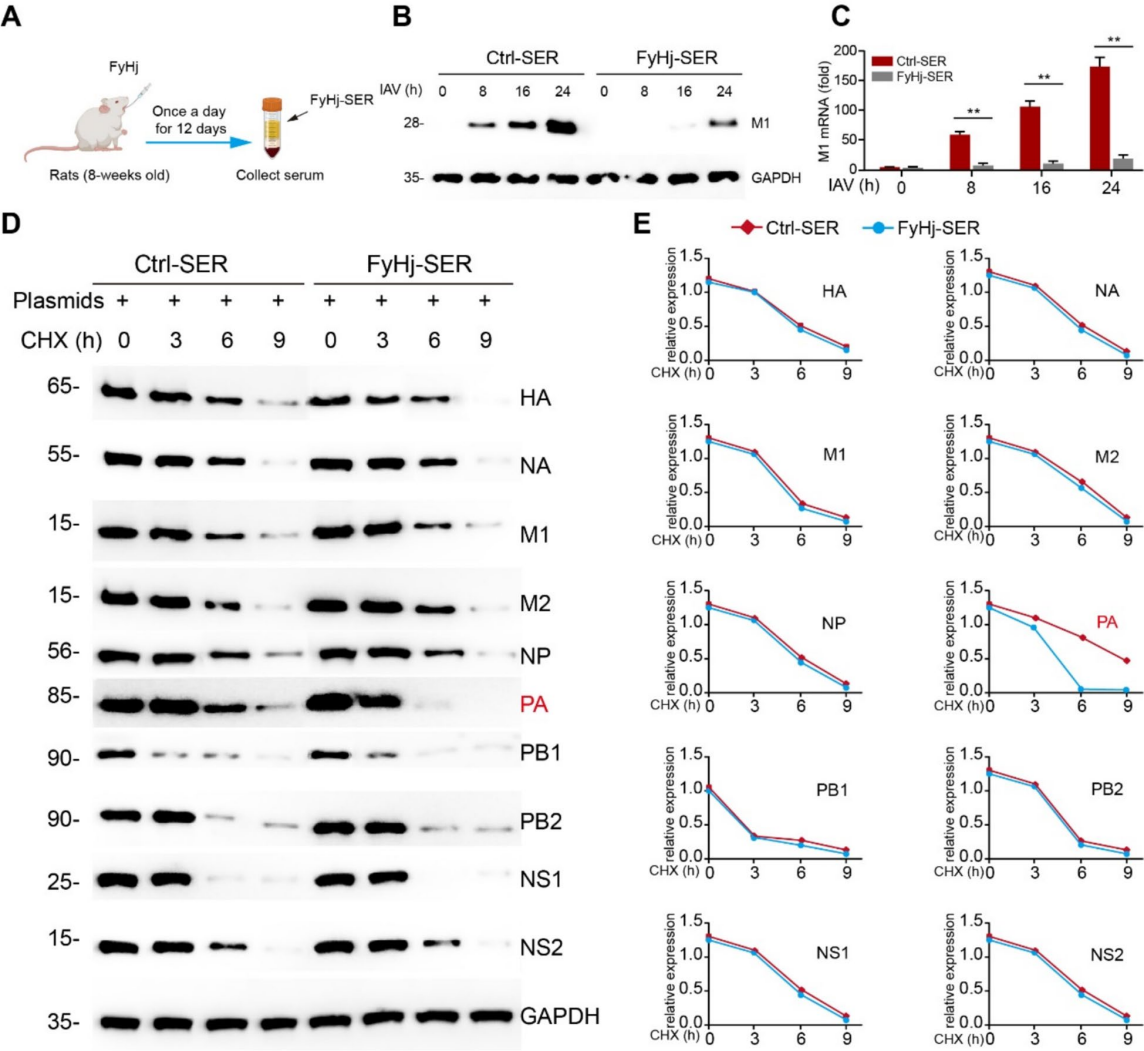
FyHj-SER inhibits the influenza virus by promoting the degradation of the PA of the influenza virus.** A** The schematic diagram of the preparation process of FyHj-SER. **B** Immunoblot analysis of M1 in A549 cells infected with an IAV multiplicity of infection (MOI) of 0.05 and then treated with serum or serum from FyHj-treated rats (FyHj-SER) for the indicated time periods. **C** qPCR of M1 in A549 cells infected with an IAV MOI of 0.05 and then treated with serum or FyHj-SER for the indicated time periods. **D** Immunoblot analysis of the indicated protein in 293 T cells transfected with each plasmid encoding hemagglutinin (HA), neuraminidase (NA), M1, M2, nuclear protein (NP), PA, polymerase basic protein 1 (PB1), polymerase basic protein 2 (PB2), NS1, and NS2 and treated with serum or FyHj-SER for 24 h. **E** The relative expression levels of HA, NA, M1, M2, NP, PA, PB1, PB2, NS1, and NS2 are quantified. Data are analyzed using Student’s *t*-test (two-tailed) and expressed as means ± standard errors of the mean. n = 5/group. **p < 0.01

The results of network pharmacology analysis indicated that numerous RNA virus-related pathways were significantly enriched by KEGG. Therefore, we first verified the direct antiviral effect of the drug. The effect of the TCM compound may be due to its becoming active after metabolism in the body. Therefore, we collected the serum from rats after they orally ingested FyHj for 12 days (once a day), and then prepared serum from FyHj-treated rats (FyHj-SER) for in vitro experiments (Fig. 2A). In vitro anti-virus assay, A549 cells infected with IAV and then treated with serum or FyHj-SER for the indicated time periods. The results of WB and qPCR showed that the viral M1 protein and mRNA expression in the control group increased over time, but in the group treated with FyHj-SER was significantly inhibited (Fig. 2B, C). We further explored the specific mechanism of its action on viral replication. All the proteins involved in influenza replication (HA, NA, M1, M2, NP, PA, PB1, PB2, NS1, and NS2) were scanned by respectively transfecting plasmids into HEK293 T cells and treated with FyHj-SER to detect the expression levels of the proteins. Interestingly, of all proteins, only PA degradation was significantly accelerated by the FyHj intervention (Fig. 2D, E). Furthermore, to investigate whether FyHj exerts direct antiviral activity, we performed a verification experiment by directly treating cells with FyHj (10 µg/mL). The results showed that this treatment produced comparable effects to those observed in the FyHj-SER group, indicating that FyHj contains components with direct antiviral activity (Figure S1). The KEGG analysis results from the previous network pharmacology study indicated that this drug target was involved in numerous RNA virus signaling pathways. Moreover, the PA protein is involved in the replication process of the influenza virus genome. Therefore, this drug suggests that it might directly intervene in the replication enzymes of RNA viruses to exert antiviral activity, providing hypotheses and references for the expansion of this formula's application to other viruses.

### FyHj-SER promotes PA degradation through interfering UBP11-mediated K48 deubiquitination

**Fig. 3:**
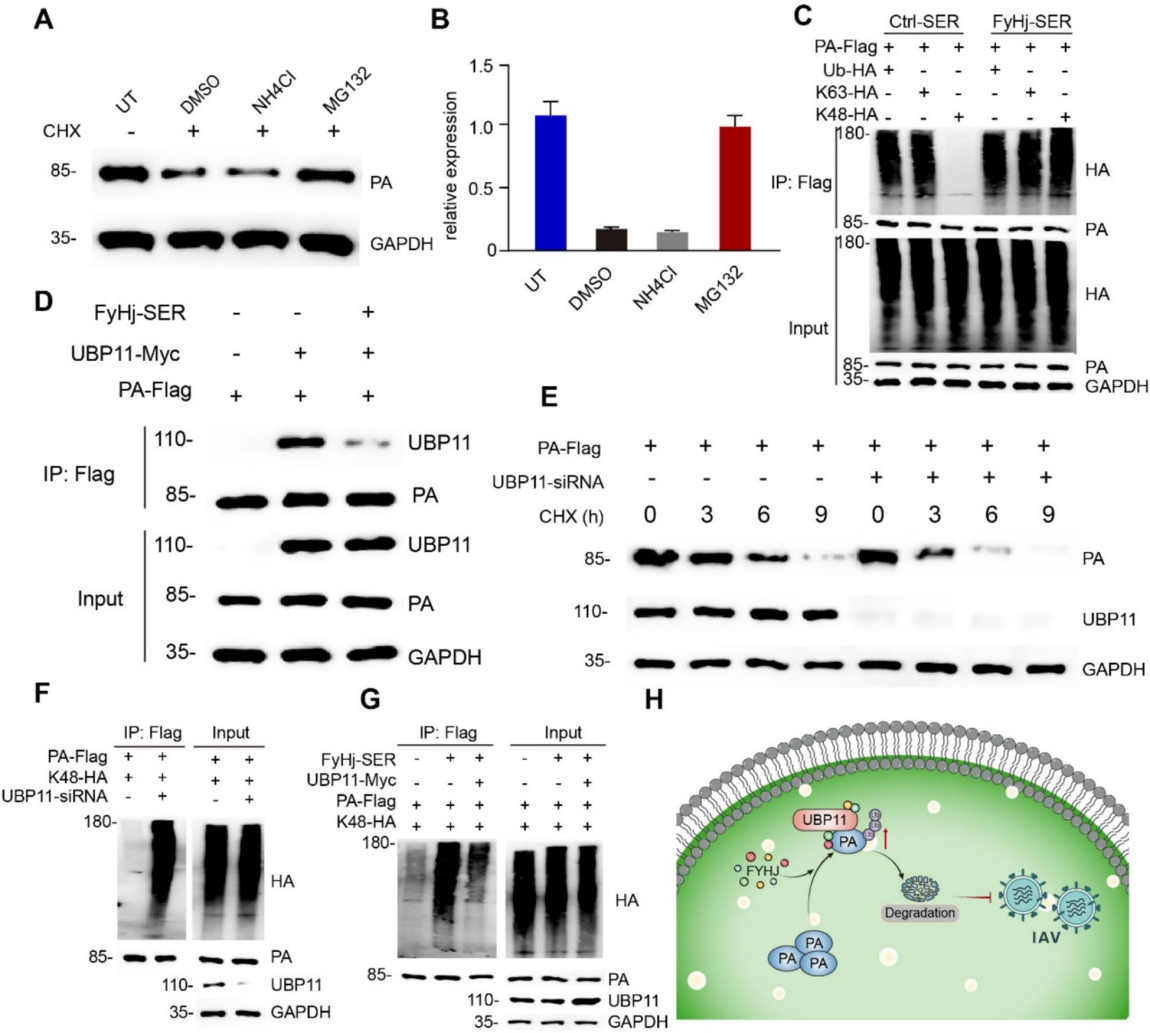
FyHj-SER PA degradation through interfering UBP11-mediated K48-linked deubiquitination.** A** Immunoblot analysis of PA in 293 T cells transfected with PA-flag for 24 h and then treated with dimethyl sulfoxide (DMSO), NH_4_Cl, or MG132, along with cycloheximide (100 μg/mL) for 9 h. **B** The relative expression levels of PA are quantified. **C** Immunoblot analysis of the indicated proteins in the immunoprecipitated samples of 293 T cells transfected with various combinations of plasmids and treated with serum or FyHj-SER for 24 h. **D** Immunoblot analysis of the indicated proteins in the immunoprecipitated samples of 293 T cells transfected with various combinations of plasmids and treated with or without FyHj-SER for 24 h. **E** Immunoblot analysis of the indicated proteins in 293 T cells transfected with Flag-PA or small interfering ribonucleic acid (siRNA) of UBP11 and treated with cycloheximide (100 μg/mL) for the indicated times, in the absence or presence of FyHj-SER. **F** Immunoblot analysis of the indicated proteins in the immunoprecipitated samples of 293 T cells transfected with various combinations of plasmids and siRNA for 24 h. **G** Immunoblot analysis of the indicated proteins in the immunoprecipitated samples of 293 T cells transfected with various combinations of plasmids and treated with or without FyHj-SER for 24 h. **H** Mechanism diagram of the inhibition of influenza virus by FyHj

Through GO analysis in network pharmacology, we found that the MF process related to ubiquitin protein ligase binding was significantly enriched. Moreover, in vitro data had confirmed that the expression level of PA protein decreases after treatment with FyHj-SER. Therefore, we further attempted to verify whether the reduction in PA protein expression is related to the ubiquitination of PA protein. By inhibiting the lysosomal degradation pathway with NH_4_Cl and ubiquitin–proteasome degradation pathway with MG132, we found that PA degradation was significantly reduced by MG132 (Fig. 3A, B), suggesting that PA was mainly degraded by the ubiquitin–proteasome pathway. Therefore, we further explored the mechanism by which FyHj-SER regulates the ubiquitin degradation of PA. We found that K48-linked ubiquitination of PA was significantly inhibited by FyHj-SER (Fig. 3C), suggesting that FyHj inhibited the IAV replication pathway through K48-mediated ubiquitin degradation of PA.

Furthermore, we explored the underlying mechanism of PA degradation and searched for potential E3 ubiquitin ligases. However, we did not find any E3 ligases that could be influenced by FyHj-SER (data not shown). Thus, we searched for deubiquitinating enzymes that may be related to its degradation. Screening by co-immunoprecipitation showed that the deubiquitinating enzymes UBP11 and PA have direct effects, which could be suppressed by FyHj-SER (Fig. 3D and Figure S2). To determine the role of UBP11, we found that knockdown of UBP11 by small interfering RNA (siRNA) significantly increased the degradation (Fig. 3E) and K48-linked ubiquitination (Fig. 3F) of PA. In addition, overexpression of UBP11 partially inhibited the effect of FyHj-SER on promoting K48-linked ubiquitination of PA (Fig. 3G). These results suggest that the FyHj drug interfered the interaction between PA and UBP11 to enhance the K48-linked ubiquitination and degradation of PA to inhibit the replication of the flu virus (Fig. 3H).

### FyHj-SER promotes the RIG-I/MAVS signal pathway and type I IFN expression

**Fig. 4:**
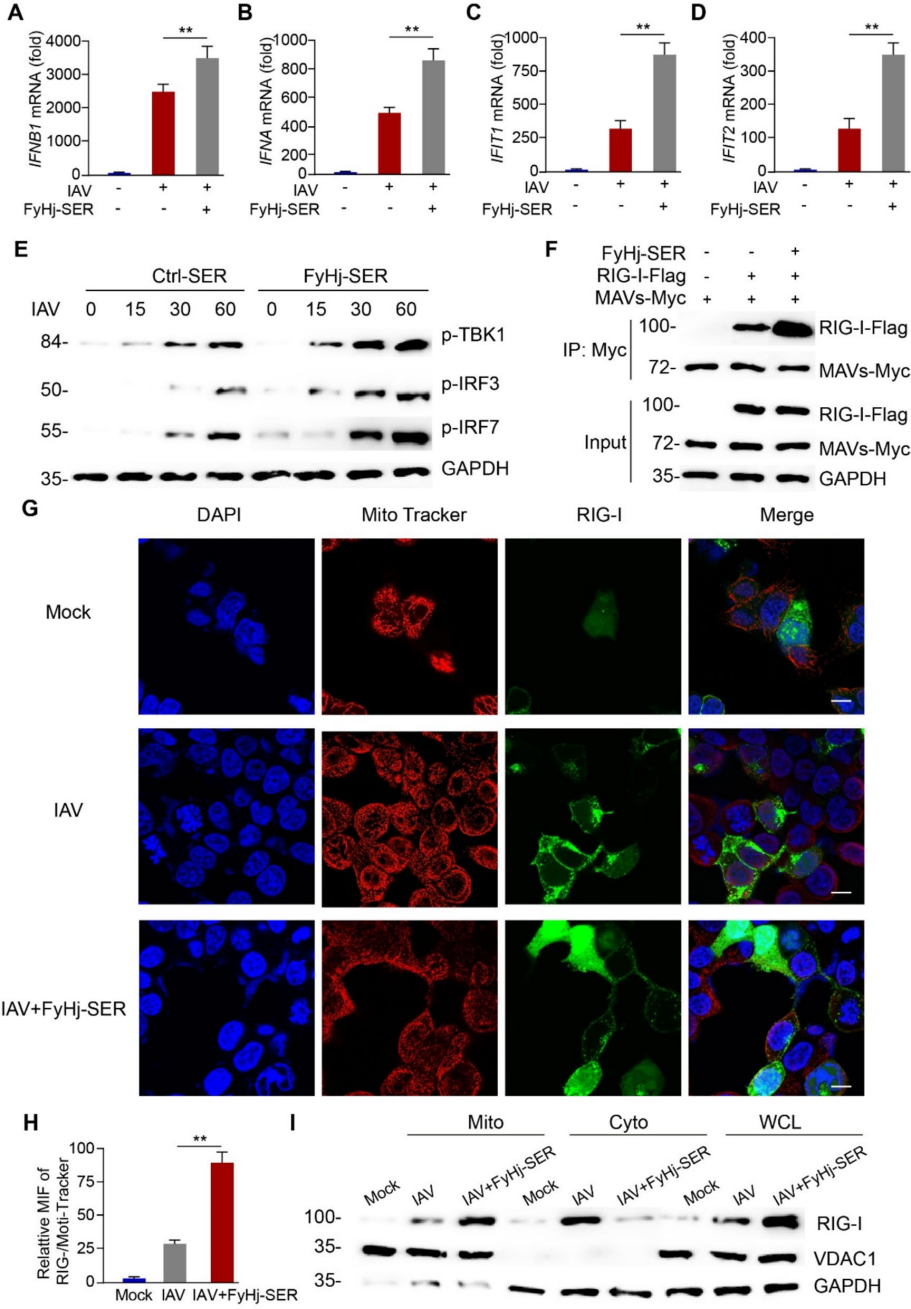
FyHj-SER promotes type I interferon (IFN) expression and the RIG-I/MAVS signal pathway up-regulation.** A**–**D** qPCR analysis of *IFNB1* (**E**), *IFNA* (**F**), *IFNT1* (**G**), and *IFNT2* (**H**) mRNA expressions from A549 cells infected with an IAV multiplicity of infection (MOI) of 0.05 in the absence or presence of FyHj-SER for 24 h. **E** Immunoblot analysis of the phosphorylation of the indicated proteins in A549 cells infected with an IAV MOI of 0.05 in the absence or presence of FyHj-SER for the indicated time. **F** Immunoblot analysis of the indicated proteins in the immunoprecipitated samples of 293 T cells transfected with various combinations of plasmids and treated with or without FyHj-SER for 24 h. **G** Confocal microscopy of endogenous RIG-I in A549 cells stained with Mito Tracker after IAV infection or mock infection in the absence or presence of FyHj-SER for 6 h. Scale bars, 10 μm. **H** The relative MIF of RIG-I and Mito Tracker are analyzed. **I** Immunoblot analysis of lysates in A549 cells after IAV infection or mock infection in the absence or presence of FyHj-SER for 6 h, followed by mitochondrial–cytoplasm extraction. Student’s *t*-test (two-tailed) and expressed as means ± standard errors of the mean. n = 5/group. **p < 0.01

The results above suggested that FyHj-SER could directly inhibit viral replication by promoting PA degradation. Also, the KEGG analysis in network pharmacology revealed that the RIG-I signaling pathway was significantly enriched. Because the components of TCM mixture are complex, the effect may not be single. Therefore, we further observed the effect of FyHj-SER on antiviral innate immunity. Firstly, we detected the expressions of I IFN (*Ifnb1* and *Ifna*) and ISG (*Ifit1* and *Ifit2*) in IAV-infected A549 cells. The qPCR result showed that FyHj-SER significantly increased the expressions of *Ifnb1* (Fig. 4A), *Ifna* (Fig. 4B), *Ifit1* (Fig. 4C), and *Ifit2* (Fig. 4D). The results suggested that the FyHj can increase I IFN expression and initiate antiviral immune response.

Therefore, we further analyzed how the FyHj drug regulates IFN gene expression. As expected, FyHj-SER could enhance the phosphorylation of TBK1, IRF3, and IRF7 related to RIG-I signal pathway (Fig. 4E). Based on this finding, we further analyzed the upstream signal of TBK1 activation induced by FyHj-SER. RIG-I is a conserved cytoplasmic pattern recognition receptor that detects viral RNA during infection and activates a type I IFN-mediated antiviral immune response. RIG-I has an N-terminal tandem caspase-activated recruitment domain that interacts with the MAVS protein and activates the antiviral signaling pathway, and this receptor–junction interaction can lead to the activation of TBK1. Our data showed that FyHj-SER could significantly enhance the interaction between RIG-I and MAVS (Fig. 4F). Furthermore, we found that FyHj-SER enhances recruitment of RIG-I to mitochondria by immunofluorescence (Fig. 4G, H) and Western blot analysis (Fig. 4I). These results suggested that FyHj drug increased the antiviral innate immunity by regulating the RIG-I/MAVS signaling pathway.

### FyHj-SER stabilizes MAVS by inhibiting TRIM25-mediated K48-linked ubiquitination of MAVS

**Fig. 5:**
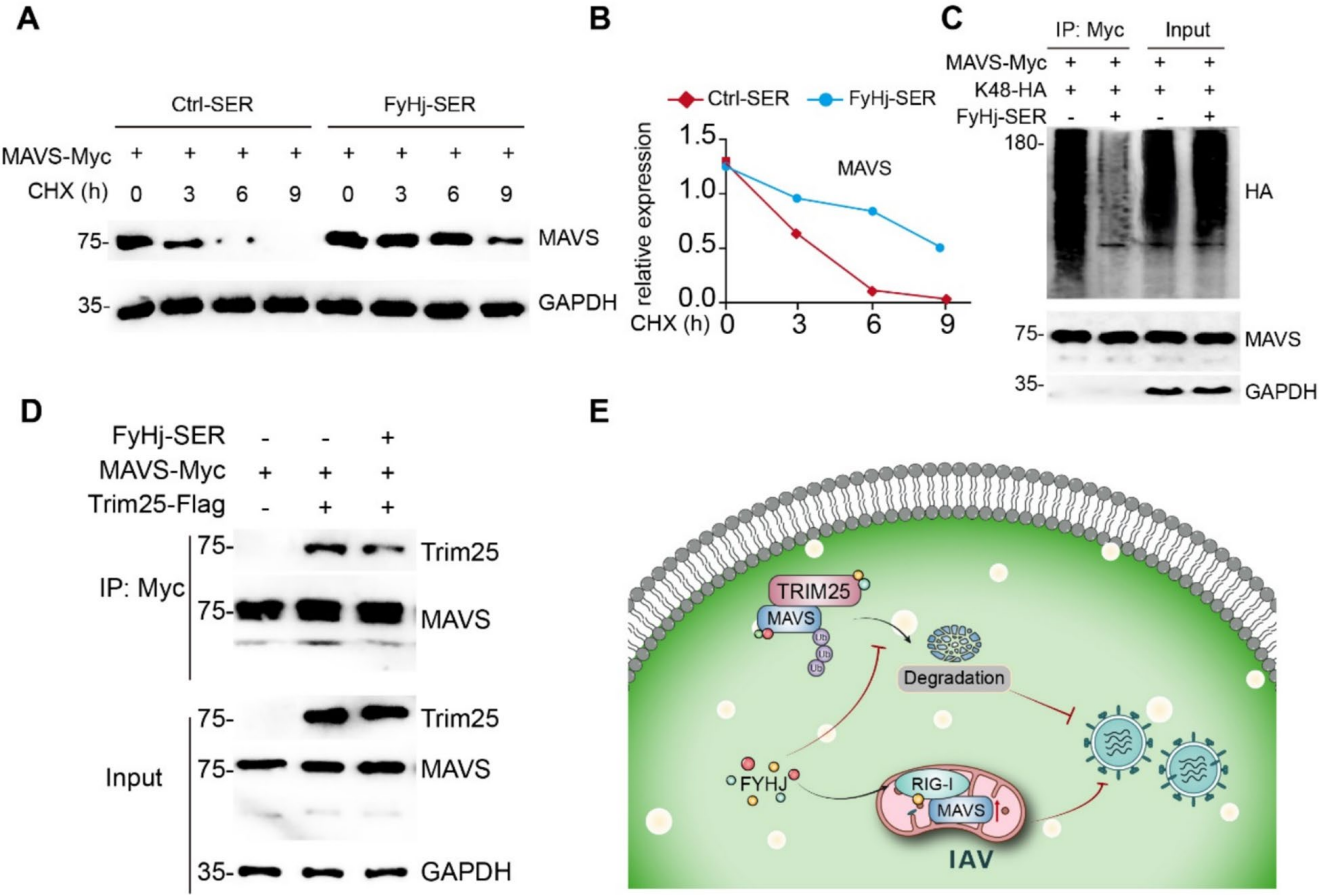
FyHj-SER inhibits MAVS K48-linked ubiquitination.** A** Immunoblot analysis of the indicated protein in 293 T cells transfected with MAVS-Myc plasmid for 24 h and treated with serum or FyHj-SER and cycloheximide. **B** The relative expression levels of MAVS are quantified. **C** Immunoblot analysis of the indicated proteins in the immunoprecipitated samples of 293 T cells transfected with various combinations of plasmids and treated with or without FyHj-SER for 24 h. **D** Immunoblot analysis of the indicated proteins in the immunoprecipitated samples of 293 T cells transfected with various combinations of plasmids and treated with or without FyHj-SER for 24 h. **E** Mechanism diagram of the inhibition of influenza virus by FyHj

As we have recently demonstrated, FyHj-SER promotes the recruitment of RIG-I to mitochondria and binds to MAVS on mitochondria to initiate an antiviral immune response. Interestingly, through the protein degradation experiment, we found that compared with the control group, MAVS degradation significantly decreased after the addition of FyHj-SER, suggesting that FyHj-SER inhibits MAVS degradation (Fig. 5A, B). Therefore, we further analyzed the possible ways that FyHj-SER affected MAVS degradation, and the results showed that the K48-linked ubiquitination of MAVS was significantly reduced under the intervention of FyHj-SER (Fig. 5C), suggesting that FyHj-SER inhibited the K48-mediated MAVS degradation. TRIM25 is an established E3 ligase for MAVS K48-linked ubiquitination and degradation. We then determined whether FyHj-SER can regulate the binding between MAVS and TRIM25 and found that the binding of MAVS and TRIM25 was significantly reduced by FyHj-SER treatment, suggesting that FyHj-SER can reduce MAVS degradation by inhibiting the binding of MAVS and TRIM25 (Fig. 5D). In summary, these results suggested that FyHj drug enhances the antiviral innate immunity by upregulating the RIG-I/MAVS signaling pathway and stabilizes MAVS by inhibiting TRIM25-mediated K48-linked ubiquitination of MAVS to exert antiviral effect (Fig. 5E).

### FyHj inhibited the viral replication and inflammatory response caused by IAV infection in mice

**Fig. 6:**
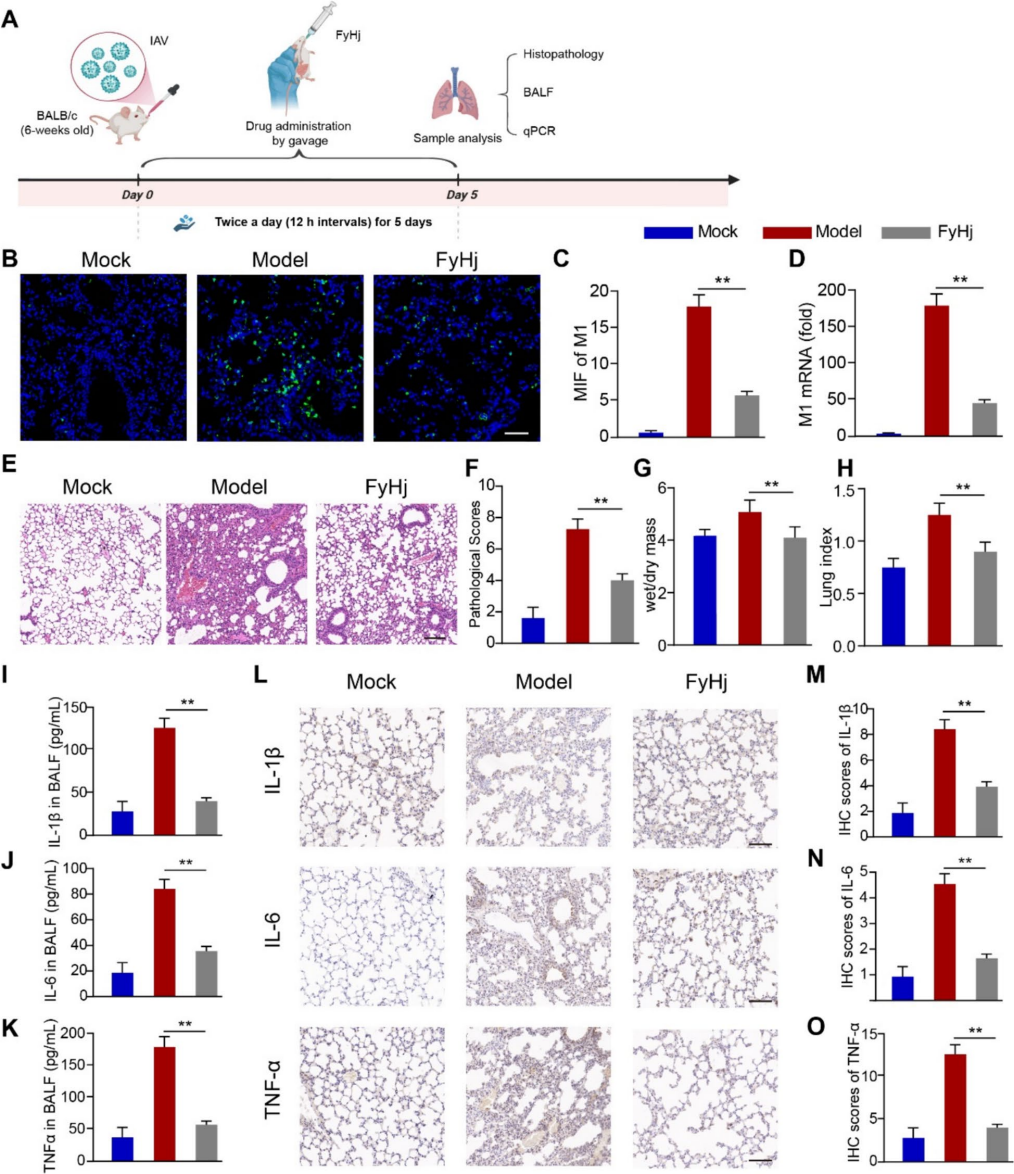
FeiyanHeji (FyHj) ameliorates pulmonary pathological injury and inflammation induced by IAV.** A** Experimental procedure for treating influenza with fyhj drug in mice. Wild-type mice are infected with IAV (3,000 PFU/animal) and treated by phosphate-buffered saline or FyHj for 5 days. **B** Immunofluorescence histochemistry analysis of M1 of IAV in the lungs of mice. Scale bars, 200 μm. **C** The mean intensity of fluorescence (MIF) of M1. **D** Quantitative polymerase chain reaction (qPCR) analysis of M1 of IAV in the lungs of mice. **E** Hematoxylin and eosin staining of the lungs. Scale bar, 200 μm. **F** Pathological scores of the lung tissue. **G** Pulmonary wet and dry mass. **H** Lung index (lung/body weight). ELISA analysis of the content of inflammatory factors of IL-1β (**I**), IL-6 (**J**), and TNF-α (**K**) in BALF. **L** Immunohistochemical staining of interleukin (IL)−1β, IL-6, and tumor necrosis factor (TNF)-α in the lungs using mouse monoclonal anti-IL-1β, anti-IL-6, and anti-TNF-α. Scale bar, 100 μm. Immunohistochemical score of IL-1β (**M**), IL-6 (**N**), and TNF-α (**O**). Data are analyzed using Student’s *t*-test (two-tailed) and expressed as means ± standard errors of the mean. n = 5/group. **p < 0.01

To investigate the effect of FyHj treatment on IAV-induced pneumonia, mice were infected with IAV and treated by FyHj for 5 days (twice a day) (Fig. 6A). After treatment, immunofluorescence histochemistry of mice lung tissue demonstrated a significant reduction in influenza M1 protein in the treatment group, while abundant fluorescence signals were detected in the PBS group (Fig. 6B, C). qPCR analysis of influenza viral M1 RNA yielded consistent the result of IFHC (Fig. 6D). The severity of lung inflammation was assessed by hematoxylin and eosin staining and pathological scores. FyHj treatment relieved IAV-induced lung histopathological damage (Fig. 6E, F). In addition, the changes in the wet and dry mass (Fig. 6G) and lung index (Fig. 6F) were similar to the lung pathological scores. Furthermore, we detected the levels of inflammatory cytokines including interleukin IL-1β, IL-6, and tumor necrosis factor (TNF)-α in bronchoalveolar lavage fluid (BALF) and lung tissues. FyHj decreased the levels of IL-1β (Fig. 6I), IL-6 (Fig. 6J), and TNF-α (Fig. 6K) in BALF. The immunohistochemical (IHC) analysis results demonstrated that the trends of inflammatory factors in the lung tissue were consistent with the findings from the BALF (Fig. 6L–O). Taken together, these results show that FyHj could ameliorate IAV-induced pulmonary inflammatory response and lung injury.

## Discussion

During an influenza season, the death rate caused by influenza pneumonia is up to 11.1% in the United States [20]. There are still no effective drugs to deal with the continuous mutations of the flu virus. “Maxingshigan Decoction” has been a clinical treatment of pneumonia for thousands of years in China, and its efficacy and safety have been confirmed by a large number of studies [21, 22]. During the period of novel coronavirus infection, “Maxingshigan Decoction” was widely used for patients with novel coronavirus pneumonia, and there were several reports in our country and abroad [23]. FyHj is a summary of the valuable experience of Professor Pei, a famous Chinese doctor, in the clinical treatment of pediatric pneumonia, especially viral pneumonia, for decades. It is based on “Maxingshigan Decoction,” on the basis of the characteristics of children’s medication to be adjusted, the clinical treatment of children viral pneumonia has been decades, the efficacy of patients affirmed. It is of great value and significance to study the mechanism of treatment of viral pneumonia. In this study, we aimed to determine whether the FyHj can inhibit viral replication and promote an antiviral natural immune response in IAV-induced pneumonia. We found that FyHj prevented IAV mRNA replication during IAV infection by inhibiting K48-linked deubiquitination of PA. Moreover, a natural antiviral immune response can be initiated by activating the RIG-I/MAVS pathway.

IAV is a negative-sense RNA virus that can replicate mRNA from the viral genome via RNA polymerase. The influenza virus genome is also known as ribonucleoprotein complex 2, which is transcribed and replicated by heteromeric viral polymerase composed of PA, PB1, and PB2. Mature viral particles have a protein envelope [24]. The envelope contains HA and NA. HA proteins bind to the airway or alveolar epithelium and trigger endocytosis of viral particles [25]. Viral HA fuses with the endosome membrane and activates the M2 ion channel, transporting it to the nucleus for viral replication [26]. Current drugs for the treatment of IAV infection inhibit viral NA, including oseltamivir and zanamivir [27] but only in the early stages of infection; therefore, the search for effective drugs to inhibit IAV replication has important research value. In this study, we found that FyHj treatment can significantly inhibit the pathological injury of lung tissue in mice. Through further study, it was found that FyHj can inhibit IAV replication by promoting PA degradation.

The lysosomal and ubiquitin–proteasome pathways are the main pathways of protein degradation, and the linkage of ubiquitin chain to protein is a multistep process involving three different types of enzymes. Ubiquitin has seven lysine residues (K6, K11, K27, K29, K33, K48, and K63) and an N-terminal methionine that can be used to form multiubiquitin chains [28]. Different types of connections are often associated with different cellular functions, and K48-linked polyubiquitin chains are involved in proteasome degradation. Through the aforementioned experiments, we found that FyHj can promote PA degradation and proved that the degradation pathway of PA is mainly the ubiquitin–proteasome pathway; however, we did not find the ubiquitin enzyme that interacts with PA in this process. Nonetheless, we found that PA can directly bind to the deubiquitinating enzyme UBP11, and under the action of drug-containing serum, the degradation of the deubiquitinating enzyme UBP11 of the K48 link significantly increased, which led to the increase in the ubiquitin degradation of PA.

In this study, we conducted a network pharmacology analysis of FyHj and used the results to guide our research on its mechanism. Our study found that, on the one hand, FyHj can inhibit viral replication by promoting PA degradation and, on the other hand, it can accelerate virus clearance by promoting the antiviral immune response of the body. Host cells have formed multiple branches of the innate immune system to defend against IAV infection, including the activation of the IFN system. After viral infection, the body first initiates an effective antiviral response, recognizes RIG-I to specifically recognize viral short-strand RNA, and then activates the IFN signal, promoting the transcription of IFN-stimulated genes [29]. The N-terminal of RIG-I transmits upstream signaling to the MAVS protein to activate type I IFN [30]. We found that FyHj drug-containing serum can promote RIG-I recruitment to mitochondria, activate type I IFN through the RIG-I/MAVS pathway, and further enhance the antiviral immune response by inhibiting MAVS ubiquitin degradation.

Finally, our study contributes to the application of FyHj in the treatment of IAV-induced pneumonia. This study provides an experimental basis for the role of FyHj in the treatment of IAV-induced pneumonia. Future studies should reveal the specific antiviral and immune mechanisms of FyHj in the affected population to promote the wider application of FyHj in clinical practice.

## Conclusions

In summary, our study showed that, on the one hand, FyHj inhibited PA deubiquitination by promoting the degradation of the deubiquitination enzyme UBP11 of the K48 link and increased its ubiquitin degradation, thus producing the effect of inhibiting viral replication, and on the other hand, FyHj promoted RIG-I recruitment to mitochondria, activated type I IFN through RIG-I/MAVS, and further enhanced the antiviral immune response by inhibiting MAVS ubiquitin degradation. Our study provides experimental basis for the practical application of FyHj in the treatment of IAV-induced pneumonia and promotes the wider application of FyHj in clinical practice.

## Supplementary Information

Additional file1

Additional file2

## Data Availability

The data that support the findings of this study are available from the corresponding author upon reasonable request.

## Abbreviations

FyHj: Feiyan Heji (traditional Chinese medicine formula)
BP: Biological process (Gene Ontology)
CC: Cellular component (Gene Ontology)
CTD: Comparative Toxicogenomics Database
DAVID: Database for Annotation, Visualization and Integrated Discovery
DL: Drug-likeness
GO: Gene Ontology
KEGG: Kyoto Encyclopedia of Genes and Genomes
MF: Molecular function (Gene Ontology)
OB: Oral bioavailability
PPI: Protein-protein interaction
STRING: Search Tool for the Retrieval of Interacting Genes/Proteins
TCM: Traditional Chinese medicine
TCMSP: Traditional Chinese Medicine Systems Pharmacology Database
TTD: Therapeutic Target Database
A549: Human lung alveolar epithelial cell line
HEK293T: Human embryonic kidney 293 cells with SV40 T antigen
MDCK: Madin-Darby canine kidney cells
H1N1: Influenza A virus subtype H1N1 (strain A/WSN/1933)
IAV: Influenza A virus
MOI: Multiplicity of infection
PFU: Plaque-forming unit
MLD_50_: 50% mouse lethal dose
BALF: Bronchoalveolar lavage fluid
DMSO: Dimethyl sulfoxide
ELISA: Enzyme-linked immunosorbent assay
IHC: Immunohistochemistry
MIF: Mean intensity of fluorescence
qPCR / qRT-PCR: Quantitative real-time polymerase chain reaction
SD: Standard deviation
siRNA: Small interfering RNA
WB: Western blot
HA: Hemagglutinin
IFIT: Interferon-induced protein with tetratricopeptide repeats
IFN: Interferon
IRF: Interferon regulatory factor
ISG: Interferon-stimulated gene
K48: Lysine 48 (ubiquitin linkage site)
MAVS: Mitochondrial antiviral signaling protein
NA: Neuraminidase
NP: Nucleoprotein
NS: Non-structural protein
PA: Polymerase acidic protein
PB1: Polymerase basic protein 1
PB2: Polymerase basic protein 2
RIG-I: Retinoic acid-inducible gene I
TBK1: TANK-binding kinase 1
TNF: Tumor necrosis factor
TRIM25: Tripartite motif-containing protein 25
UBP11: Ubiquitin-specific protease 11
DMEM: Dulbecco’s modified Eagle’s medium
FBS: Fetal bovine serum
PBS: Phosphate-buffered saline
FyHj-SER: Feiyan Heji serum (from FyHj-treated rats)
SPF: Specific-pathogen-free
IL: Interleukin
