# Supplementary material for: FeiyanHeji multifunctionally inhibits influenza virus via PA protein degradation and RIG-I signaling pathway potentiation
Source: Chin Med. 2026 Apr 22;21:120. doi: 10.1186/s13020-026-01372-6 (PMC13101370; doi:10.1186/s13020-026-01372-6)
Supplement: Supplementary file 2 — Additional file2 [file 13020_2026_1372_MOESM2_ESM.docx]

Supplementary

FeiyanHeji multifunctionally inhibits Influenza Virus via PA Protein Degradation and RIG-I Signaling Pathway Potentiation


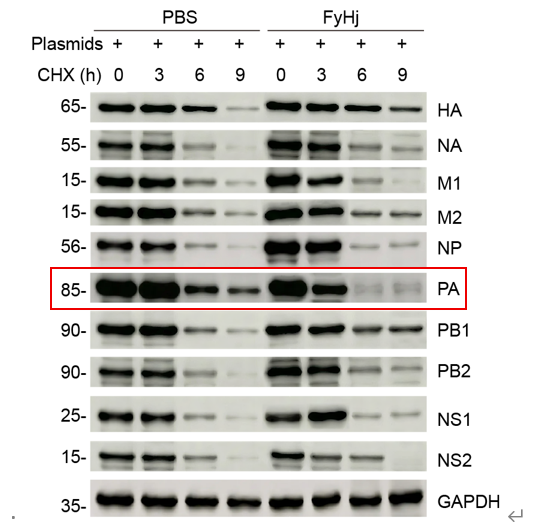


**Figure S1** Immunoblot analysis of the indicated protein in 293 T cells transfected with each plasmid encoding hemagglutinin (HA), neuraminidase (NA), M1, M2, nuclear protein (NP), PA, polymerase basic protein 1 (PB1), polymerase basic protein 2 (PB2), NS1, and NS2 and treated with PBS or FyHj for 24 h.


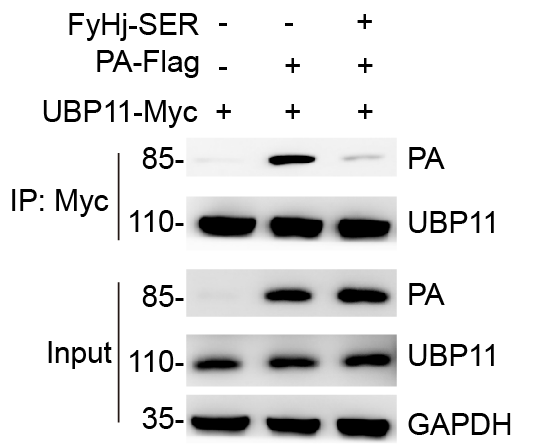


**Figure S2.** Immunoblot analysis of the indicated proteins in the immunoprecipitated samples of 293 T cells transfected with various combinations of plasmids and treated with or without FyHj-SER for 24 h.
